# Transcranial direct-current stimulation enhances Pavlovian tendencies during intermittent loss of control

**DOI:** 10.3389/fpsyt.2023.1164208

**Published:** 2023-05-09

**Authors:** Terezie Sedlinská, Lara Bolte, Eirik Melsæter, Matthias Mittner, Gábor Csifcsák

**Affiliations:** ^1^Department of Psychology, UiT The Arctic University of Norway, Tromsø, Norway; ^2^Psychiatric University Hospital, Zürich, Switzerland

**Keywords:** Pavlovian bias, controllability, learned helplessness, mid-frontal theta power, tDCS, medial prefrontal cortex

## Abstract

**Introduction:**

Pavlovian bias is an innate motivational tendency to approach rewards and remain passive in the face of punishment. The relative reliance on Pavlovian valuation has been found to increase when the perceived control over environmental reinforcers is compromised, leading to behavior resembling learned helplessness (LH).

**Methods:**

Sixty healthy young adults underwent a Go-NoGo reinforcement learning task and received anodal high-definition transcranial direct current stimulation (HD-tDCS) over the medial prefrontal/dorsal anterior cingulate cortex in our randomized, double-blind, sham- controlled study. Furthermore, we evaluated changes in cue-locked mid-frontal theta power derived from simultaneous electroencephalography (EEG). We hypothesized that active stimulation would reduce Pavlovian bias during manipulation of outcome controllability, and the effect would be accompanied by stronger mid-frontal theta activity, representing arbitration between choice strategies in favor of instrumental relative to Pavlovian valuation.

**Results:**

We found a progressive decrease in Pavlovian bias during and after loss of control over feedback. Active HD-tDCS counteracted this effect while not affecting the mid-frontal theta signal.

**Discussion:**

The results were at odds with our hypotheses but also with previous findings reporting LH-like patterns during and after loss of control without brain stimulation. The discrepancy may be related to different protocols for the controllability manipulation. We argue that the subjective evaluation of task controllability is crucial in mediating the balance between Pavlovian and instrumental valuation during reinforcement learning and that the medial prefrontal/dorsal anterior cingulate cortex is a key region in this respect. These findings have implications for understanding the behavioral and neural underpinnings of LH in humans.

## 1. Introduction

The ability to learn from the environment and to act in an adaptive way is a crucial feature of all living organisms. Multiple computational mechanisms of environmental feedback integration have been identified in the behavior of humans and non-human animals. These include various modes of learning from rewards and punishments (reinforcement learning; RL), such as Pavlovian and instrumental valuation (1, 2). Both of these mechanisms rely on an identification of a temporally stable situation (stimulus) and its association with a positively or a negatively valued event (outcome). Instrumental valuation assumes the combination of stimuli and the organism’s actions to cause an outcome, whereas Pavlovian valuation takes into account only the stimulus-outcome pairing and disregards the organism’s actions (3–5). Even though the Pavlovian system caches only past outcomes and not the organism’s actions, it produces a motivational bias toward approaching and consuming potential rewards and remaining passive in the face of punishment-predictive cues, Pavlovian bias (PB) (6, 7). This can be advantageous in situations where a fast, automatic reaction is required but can also create a conflict with more flexible ways of learning (4, 8, 9).

Adaptive organisms rely on both valuation systems, and the way Pavlovian-instrumental interactions influence behavior strongly depends on the perceived controllability of the situation (10, 11). Healthy humans have been found to rely more heavily on Pavlovian valuation when the control over environmental reinforcers is compromised (7, 10, 12–14). When perceived control does not match the actual degree of controllability of the environment, the agents can underestimate the influence of actions and can become overly passive. This may result in behavior resembling learned helplessness (LH) (15). In animal models, LH can be elicited by a lack of contingency between actions and environmental feedback. When control is regained, the animals fail to adequately update their perception of controllability and continue being passive, manifesting in a “transfer effect.” As LH can produce anxiety, motor passivity, and impair decision-making, it has been used as a laboratory model of depression (15, 16).

Recently, the “learned controllability” account of LH has been proposed, according to which LH-like behavior is not induced by learned uncontrollability, but rather, it is an expression of an innate response tendency to remain passive in face of possible punishment (15). Conversely, once the agent experiences control over the environment, suppression of such inaction tendencies is triggered, leading to active exploration and successful coping behavior (15). Interestingly, passivity during the anticipation of aversive events belongs to the repertoire of the Pavlovian valuation system (4, 7, 17), and hence it is feasible that learned action-outcome contingency triggers top-down mechanisms to inhibit automatic Pavlovian response tendencies in favor of instrumental valuation (14).

Perceived controllability has been associated with activity in the medial prefrontal cortex (mPFC) and the dorsal anterior cingulate cortex (dACC), linking these regions to LH (11, 15, 18, 19). In line with the learned controllability account, animal studies indicate that low perceived controllability downregulates the mPFC, which, in turn, disinhibits subcortical structures implicated in LH-like behavior (15, 18, 20–22). Further, studies in humans provided evidence that theta-band (4–8 Hz) oscillatory activity recorded above the mPFC/dACC correlates with trial-by-trial fluctuations in subjectively inferred controllability during RL (13). Enhanced frontal midline theta (FMθ) power has also been observed prior to overriding PB in favor of instrumental valuation in Pavlovian vs. instrumental conflict situations, such as those requiring withholding actions for reward-predicting cues (17, 23, 24). These results align well with the learned controllability theory by Maier and Seligman (15), based on which one can expect that FMθ reflects the arbitration between the Pavlovian and instrumental valuation during intermittent absence of outcome controllability in an RL task, an assumption that gained support recently (14).

Altogether, the mPFC/dACC seems to be crucial for mediating both Pavlovian-instrumental interaction and the effect of outcome controllability on task performance. In line with this assumption, healthy humans receiving anodal high-density transcranial direct current stimulation (HD-tDCS) over the mPFC/dACC while facing uncontrollable outcomes during RL improved task performance after control was regained. In addition, PB was reduced in participants receiving active HD-tDCS during diminished outcome controllability but not in participants undergoing either active HD-tDCS or the controllability manipulation alone, suggesting that the level of control over rewards/losses interferes with the degree to which mPFC/dACC suppresses PB.

The previously reported protocols for manipulating outcome controllability during RL consisted of pairing participants in terms of reward/loss frequency, similar to “yoking” paradigms in animal studies of LH (14, 15). Despite the observed effects on PB and response accuracy, these attempts to translate yoking protocols from animals to humans failed to induce behavioral effects characteristic of LH since they were not accompanied by a subjective feeling of loss of control and lacked a transfer effect between the blocks (i.e., worse task performance in blocks with regained control). A study using a different LH-induction paradigm found a reward frequency manipulation to be more potent than a pure contingency manipulation in inducing the desired behavioral changes (25). Therefore, we abandoned the pairwise yoking design in this study and introduced a block where outcomes are delivered entirely at random so that learning becomes impossible and reward frequency is lower (and loss frequency higher) in comparison to the controllable blocks. We expected our new protocol to interfere more robustly with task performance and perceived controllability, and thus generate conditions suitable for evaluating the effect of mPFC/dACC stimulation via HD-tDCS in the context of LH-like behavior. We hypothesized that active stimulation would alleviate the unfavorable effects of loss of control and improve decision-making. In particular, by suppressing maladaptive Pavlovian response tendencies in conflict trials, participants were anticipated to show higher response accuracy both during and after active stimulation compared to those receiving sham HD-tDCS. To verify that the behavioral effects of random feedback are related to the suppression of Pavlovian response tendencies and activity in the mPFC/dACC, we recorded EEG to extract cue-locked FMθ during decision-making as a neural marker of Pavlovian-instrumental arbitration.

## 2. Methods

### 2.1. Participants

We recruited 60 healthy young adults from the university community using advertising over social media. The study could be completed in English or Norwegian based on the participant’s preference. All participants had to be over 18 years of age and meet the prespecified inclusion criteria: have no history of neuropsychiatric disease or severe somatic impairment and have good or corrected eyesight. In addition, they were asked to have had a good night’s sleep prior to the data collection and not be under the influence of any psychotropic substances except for caffeine or nicotine. All participants signed informed consent and were assigned to one of the two experimental groups (*n* = 30 in each). One researcher (GC) performed a pseudo-random group allocation, assigning five and five participants to each group randomly out of every 10 incoming participants. Group membership was blinded to both the participants and the experimenters (LB, EM, and TS).

The behavioral analysis was performed on data from 57 participants (age 24.4 ± 4.0 years, 33 female, 49 right-handed). Three participants were excluded from the analysis for not having at least 10% Go and 10% NoGo responses in each block, resulting in data from 30 participants who received active HD-tDCS (age: 24.1 ± 3.2 years, 19 female, 26 right-handed) and 27 sham stimulation (age: 24.8 ± 4.8 years, 14 female, 23 right-handed) being analyzed. Two additional participants (one from each HD-tDCS group) were excluded from the EEG data analysis due to excessive artifacts.

The detailed study protocol was approved by the ethics committee of the Department of Psychology, UiT, the Arctic University of Norway, and complied with the Declaration of Helsinki. All study materials and data are available at https://osf.io/73huk/.

### 2.2. Experimental procedure

After signing the informed consent, the participants were asked to read the task instructions carefully. The instructions were summed up orally by the experimenter, and the participants’ questions were answered. Next, the participants proceeded to a short practice session of the experimental task with a set of cards that was not used in the main task. Afterward, they completed a short quiz ensuring their understanding of the task, which was again discussed with the experimenter. Then the participants performed three blocks of the RL task, each block consisting of 160 trials (four cards, each shown 40 times in random order). At the end of each block, they were asked to rate the degree of perceived control over the feedback and their perceived success at receiving rewards on an analog visual scale ranging between 0 and 100.

In order to motivate the participants to engage in the task, they were told that they would receive a shopping voucher worth 200 NOK and additional 100 NOK if they performed above a certain (not pre-specified) threshold. Then, after three blocks of the task, all participants were told they performed well and would therefore receive the full reward of 300 NOK (roughly 35 USD) as a shopping voucher. Finally, they were asked to provide a forced choice about whether they thought they received real or sham stimulation.

### 2.3. Experimental task

We used a computerized version of an orthogonalized Go-NoGo RL task (4, 14, 17) designed to investigate PB during instrumental learning. The participants should learn to maximize the rewards and minimize losses by trial-and-error, through correctly responding (“Go”) or not responding (“NoGo”) to a given stimulus (card). Response options were framed as the choice whether one decides to “pick up” a card from the table or not during a card game.

There were four stimuli (cards) in each block: two Win cards that potentially delivered a reward of 10 points or nothing (0 points), and two Avoid cards resulting either in a loss (−10 points) or neutral feedback (0 points). The participants were also informed that each action (key press) would come with a cost of one point, mimicking the cost of real-life actions that may subtly incentivize inaction. Thus, outcomes could range between −11 and + 10 points, depending on the feedback (−10, 0, 10 points) and the presence/absence of the “go-cost” (−1 point). For each card, there was a correct response that did not change during the block resulting in two PB-congruent conditions: Cards where the correct response was an action in order to receive a reward (Go-to-Win) and passivity with the prospect of avoiding punishment (NoGo-to-Avoid), counterbalanced by two PB-incongruent conditions: NoGo-to-Win and Go-to-Avoid. A new set of cards was presented on each block, and choice strategies had to be established again via learning.

**Figure 1:**
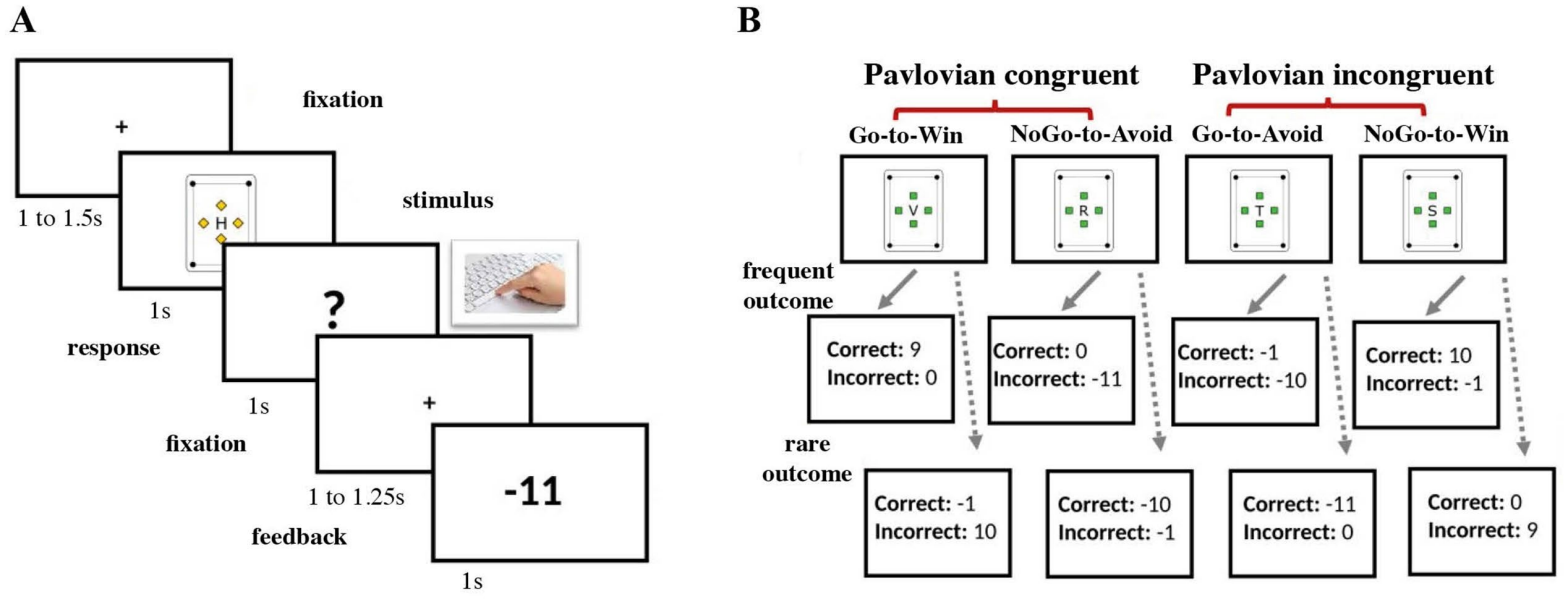
Experimental task structure. **(A)** Structure of a single trial with a fixation period, stimulus presentation, response screen, and feedback presentation. **(B)** The hidden structure of one block with four cards and their possible outcomes. In blocks 1 and 3, frequent outcomes were presented with a probability of 80% and rare outcomes with a probability of 20%, respectively. In block 2 the probability was set to 50% for each outcome.

Each trial started with a fixation cross in the middle of the screen followed by one of the four cards. After another brief fixation period, a question mark appeared on the screen, signaling the response window, during which participants could respond with a keypress. Finally, based on the card type and the response, the feedback was presented on the screen (see Figure 1A).

The participants were instructed that the rewards would be delivered stochastically with a given but unknown probability. Unbeknownst to the participants, Blocks 1 and 3 had a response-feedback contingency of 80/20%, with the optimal outcome being delivered in 80% of trials with a correct response. The rare outcome (20% of the trials) was the card’s feedback for the complementary action. In contrast with earlier studies (14, 17, 26), we decided to increase the contingency level in the controllable blocks to 80/20% (compared to the original 70/30%) in order to contrast the “control” vs. “no control” blocks even further. During the second block, rewards were delivered at random (response-feedback contingency was set to 50/50%). Therefore, the outcome was independent of the participants’ response and learning was not possible (Figure 1B).

### 2.4. EEG recording and HD-tDCS

We used an eight-channel hybrid tES/EEG Starstim device (Neuroelectrics, Barcelona, Spain) with Ag/AgCl electrodes (diameter: 12 mm), neoprene head caps, and conductive gel (SignaGel) to record EEG and deliver HD-tDCS through the same electrodes. Electrodes were placed at scalp positions Fpz, AFz, Fz, FCz, Cz, F3, F4, and TP10, while the reference/ground electrodes CMS and DRL were positioned at TP9. EEG was recorded in Blocks 1 and 3 via all scalp electrodes. In Block 2, HD-tDCS was applied with electrode Fz serving as anode (2 mA) and the surrounding electrodes Fpz, Cz, F3, and F4 as returns (0.5 mA each). Computational modeling of the cortical distribution of the tDCS-induced electric field on realistic head models (27) confirmed that this montage provides relatively focused stimulation of the mPFC/dACC (28). In addition, this montage was identical to the one used in a previous study that resulted in improved value-based choices following manipulations of reward/loss controllability for active vs. sham HD-tDCS (26). Active stimulation consisted of a 30-s ramp-up, 15 min stimulation, and 30-s ramp-down. In contrast, the sham session only contained two 30 s ramp-up/ramp-down periods at the beginning and end of a 16 min period, with no stimulation in-between. To ensure blinding during the stimulation, we applied a local anesthetic cream containing lidocaine/prilocaine (“EMLA”) under the stimulation electrodes.

#### 2.4.1. Preprocessing of the EEG data

Following our previous study (26), we analyzed cue-locked FMθ between HD-tDCS groups and experimental blocks (pre- vs. post-stimulation). First, we measured cue-locked FMθ in an *a priori* defined scalp region, time interval, and frequency range (14, 17). Following time-frequency transformation of segmented data using continuous complex Morlet wavelets (from 1 to 30 Hz in 30 linear-spaced frequency steps, Morlet parameter c = 3, baseline correction between −300 and − 200 ms), data were averaged separately for Pavlovian-congruent (Go-to-Win, NoGo-to-Avoid) and Pavlovian-conflict (Go-to-Avoid, NoGo-to-Win) trials, and two blocks (Block 1 and 3). We also collected EEG with a limited number of electrodes (AFz, FCz, and TP10) during HD-tDCS in block 2. However, these data were not analyzed due to the extensive stimulation-related artifacts. FMθ power was extracted from our frontocentral pooled channel (Fz/FCz/Cz) for each participant between 175 and 350 ms post stimulus and at 4–8 Hz (the mean of three wavelet layers with central frequencies at 5.17, 5.81, and 6.53 Hz, respectively).

### 2.5. Model-agnostic analysis

We calculated response accuracy per card type and block as the number of correct responses/total number of trials. We note that interpreting accuracy for Block 2 with random outcomes is not straightforward, as participants could not differentiate between Go and NoGo cards due to the absence of response-contingent feedback. Thus, in the absence of PB, accuracy should be around 50% for all four cards in this block. For participants with strong PB, however, accuracy should be higher than 50% for one Win and one Avoid card (those randomly labeled as Go-to-Win and NoGo-to-Avoid). In contrast, accuracy for the other two cards (NoGo-to-Win and Go-to-Avoid) should be proportionally below 50%.

We calculated a measure of the degree of PB, the Pavlovian performance index (PPI) as a mean of reward-based invigoration (RBI) and punishment-based suppression (PBS). RBI is calculated as the number of Go responses on Win trials divided by the total number of Go responses, while PBS was quantified as the proportion of NoGo responses on Avoid trials out of all NoGo trials. We compared response accuracy and PPI (as well as RBI and PBS) using repeated-measures ANOVA (rmANOVA) with Stimulation (active vs. sham) as between-subject, and Block (1, 2 and 3) as within-subject factor. We also analyzed the association of Congruency (Pavlovian-congruent vs. conflict trials) and Valence (Win vs. Avoid cards) with accuracy.

To follow up significant interactions from the rmANOVAs, we relied on robust estimation statistics. We reported effect size estimates (Cohen’s *d*), corresponding bias-corrected and accelerated 95% confidence intervals and permutation-based *p* values (5,000 bootstrap samples) instead of running conventional *post-hoc* tests (29).

FMθ power values were analyzed using rmANOVA: Block (Block 1 vs. 3) and Congruency (Pavlovian-congruent vs. conflict) were entered as within-subject factors, and Stimulation (active vs. sham) was entered as between-subject factor.

### 2.6. Computational modeling

In order to estimate the latent parameters of RL, we used the approach used in previous studies (14, 17, 23) and fitted an RL model to the behavioral data. We were primarily interested in group differences in the PB parameter *π*. Further extracted parameters represented the individual block-wise randomness of choice (temperature *β*), learning rate (*α*), and the general tendency to initiate actions (go-bias, *b*). The decision rule on whether to pick up a card was implemented as a Bernoulli experiment with the probabilities p(Go) and p(NoGo) = 1-p(Go):


$$p\left(Go|s_{t,j,i}\right)=\frac{\exp\left[W_{t}\left(Go|s_{t,j,i}\right)\beta_{j,i}^{-1}\right]}{\exp\left[W_{t}\left(Go|s_{t,j,i}\right)\beta_{j,i}^{-1}\right]+\exp\left[W_{t}\left(NoGo|s_{t,j,i}\right)\beta_{j,i}^{-1}\right]}$$

According to this equation, the probability of picking up (Go) a given card *s_t,j,i_* on a trial *t*, of participant *i* during block *j* was calculated as a softmax function, based on the “weight” *W_t_* assigned to the two possible response options. The weight *W_t_* on each trial *t* for a given action was calculated as a combination of the instrumental value *Q_t_* of the given stimulus-outcome combination, the value *V_t_* of the given stimulus scaled by the Pavlovian parameter *π*, and the participant’s go-bias *b_i,j_*.


$$W_{t}\left(a|s_{t,j,i}\right)=\{\begin{array}{l} Q_{t}\left(Go|s_{t,j,i}\right)+b_{j,i}+\pi_{j,i}V_{t}\left(s_{t,j,i}\right)\;if\;a=Go\\ Q_{t}\left(NoGo|s_{t,j,i}\right)if\;a=NoGo \end{array}$$

The value *V_t_* of a given card *s_t,j,i_* was updated at the end of each trial based on the reward *r_t_* received during that trial. The current reward *r_t,j,i_* was used to update the value by calculating the prediction error and updating the previous value weighted by the individual learning rate *α_j,i_* on a given block *j*.


$$V_{t}\left(s_{t,j,i}\right)=V_{t-1}\left(s_{t,j,i}\right)+\alpha_{j,i}\left[r_{t,j,i}-V_{t-1}\left(s_{t,j,i}\right)\right]$$

The instrumental state-action values *Q_t_(a|s)* were calculated in the same way for every action *a* (Go or NoGo) for a given card *s_t,j,i_*, resulting in eight continually updated *Q* values during a block.


$$Q_{t}\left(a|s_{t,j,i}\right)=Q_{t-1}\left(a|s_{t,j,i}\right)+\alpha_{j,i}\left[\;r_{t,j,i}-Q_{t-1}\left(a|s_{t,j,i}\right)\right]$$

Similarly as in previous studies (14), we used hierarchical Bayesian modeling using Hamiltonian Monte-Carlo algorithms (30) implemented in Stan (31). For an in-depth description of the advantages of Bayesian methods for estimating such hierarchical models, we refer to (32). We used six parallel chains with warm-up period of 1,000 samples each such that 6,000 samples were drawn from the converged chains. The trace plots for all variables were visually inspected for convergence. The Gelman-Rubin diagnostic (33) was *R^* ≤ 1.05 for all parameters.

The dependency of each model parameter on Block, Manipulation of controllability, HD-tDCS, and their interactions were included directly at the group-level in the hierarchical model. Posterior densities for the estimated coefficients were calculated and regarded as relevant if their 95% highest density interval (95% HDI) excluded zero. When reporting regression coefficients, we report posterior mean change (MC), 95% HDI and the evidence ratio (ER) in favor of a positive (ER^⊕^) or a negative effect (ER^⊖^). ER can be interpreted as an odds ratio, calculated as the ratio of two probabilities: For ER^⊕^, the probability of the effect being positive, P(b > 0), divided by the probability of the effect being zero or negative, 1-P(b > 0), or its inverse for ER^⊖^.

## 3. Results

### 3.1. Model-agnostic analysis

#### 3.1.1. Blinding success

The percentage of participants guessing their stimulation condition correctly was statistically comparable across groups [active HD-tDCS: 50%, sham HD-tDCS: 74%; χ^2^(1) = 3.47; *p* = 0.062], even though we note that more participants undergoing active stimulation guessed that they received active stimulation than in the sham group (active HD-tDCS: 15, sham HD-tDCS: 7 participants). Still, we conclude that our blinding procedure was effective in disguising the HD-tDCS condition participants were randomized to.

#### 3.1.2. Success and control

**Figure 2:**
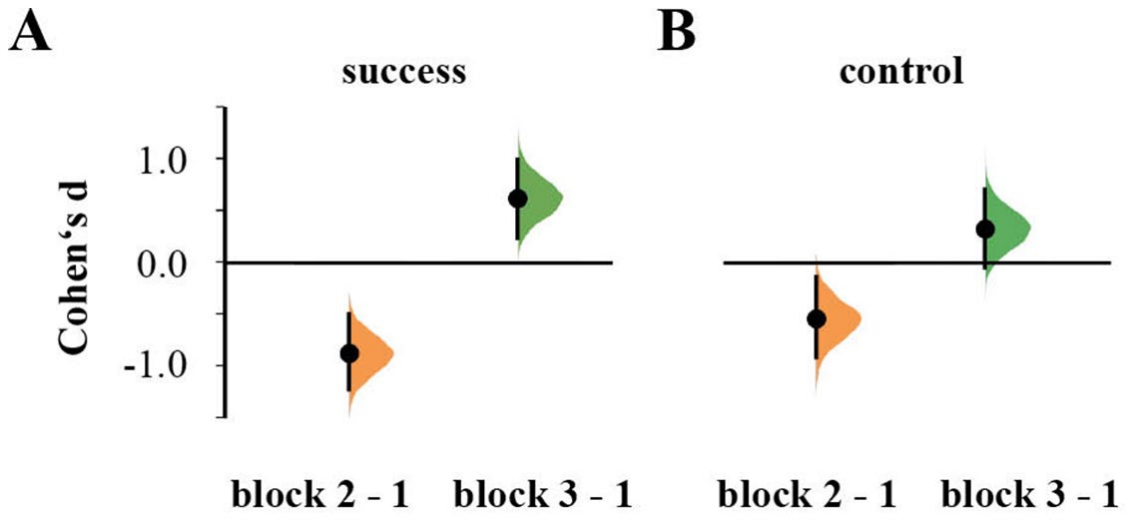
Cumming estimation plots for Cohen’s d of reported subjective levels of success **(A)** and control **(B)** during the second and third block in comparison to the first block.

Repeated-measures ANOVA showed an effect of Block on the perceived level of success [*F*(2,108) = 40.285, *p* < 0.001, η_p_^2^ = 0.427], but not of Stimulation [*F*(1,54) = 0.073, *p* = 0.788, η_p_^2^ = 0.001] or their interaction [*F*(2,108) = 2.610, *p* = 0.078, η_p_^2^ = 0.046]. Self-reported success level decreased in block 2 (*d* = −0.933, 95% CI [−1.290, −0.552]) and improved in the third block beyond the level of block 1 (*d* = 0.596, 95% CI [0.207, 0.977]; Figure 2).

Similarly, reported perceived control over feedback differed between blocks [*F*(2,108) = 17.867, *p* < 0.001, η_p_^2^ = 0.249], but not between groups [*F*(1,54) = 0.007, *p* = 0.934, η_p_^2^ = 61.291e^−4^]. It was again lowest in the second block (*d* = −0.643, 95% CI [−1.050, −0.238]) and recovered in the third block (*d* = −0.933, 95% CI [−1.240, 0.652]; Figure 2). There was no interaction between Block × Stimulation [*F*(2,108) = 1.268, *p* = 0.286, η_p_^2^ = 0.023].

#### 3.1.3. Pavlovian performance index

**Figure 3:**
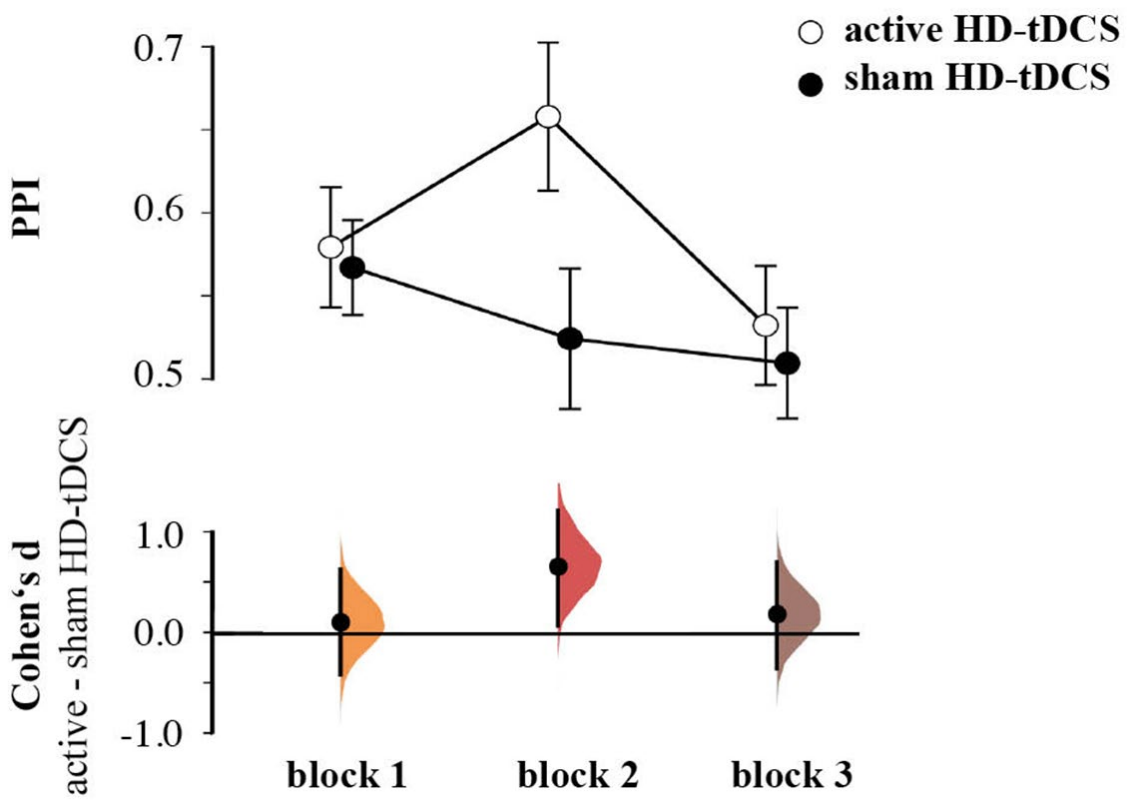
Pavlovian performance index by group and block, showing mean and 95%CI (upper panel), Cumming plots of Cohen’s d between active and sham HD-tDCS (lower panel).

For the Pavlovian performance index (PPI), the rmANOVA revealed a Stimulation effect [*F*(1,55) = 4.117, *p* = 0.047, η_p_^2^ = 0.070], as well as a significant Block × Stimulation interaction [*F*(1,55) = 3.802, *p* = 0.031, η_p_^2^ = 0.065], which was due to higher PPI during the second block for the group receiving active HD-tCDS than the sham stimulation (*d* = 0.672, 95% CI [0.081, 1.220]; Figure 3). The interaction between Block × Stimulation × Pavlovian performance index (i.e., RBI vs. PBS) was not significant [*F*(1.7395.16) = 0.997, *p* = 0.363, η_p_^2^ = 0.018]. Here, we focus on effects including Simulation. See the Supplementary Table 1 for all effects on Pavlovian performance index.

#### 3.1.4. Accuracy

**Figure 4:**
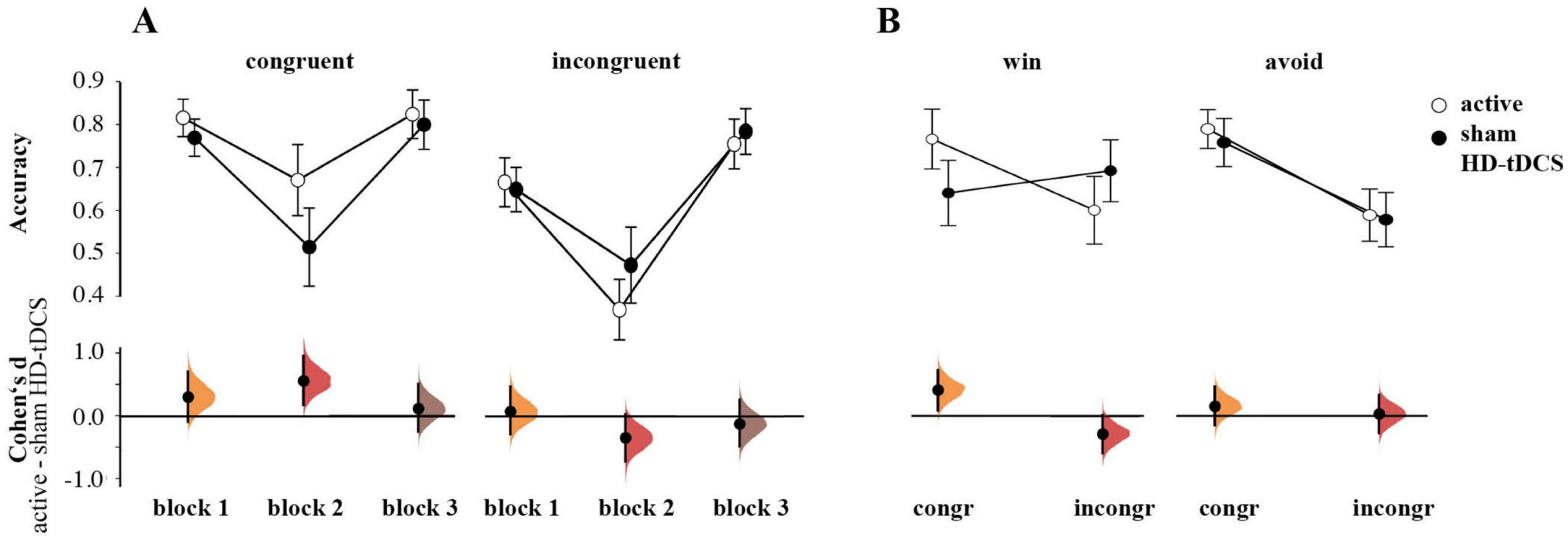
**(A)** Differences in accuracy between active HD-tDCS and sham group by blocks and congruence. **(B)** Group differences in accuracy by congruence and valence. Both plots **(A,B)** show the mean and 95%CI, as well as Cumming plots of Cohen’s *d* between active and sham stimulation.

The rmANOVA of response accuracy showed no main effect of Stimulation [*F*(1,55) = 0.509, *p* = 0.478, η_p_^2^ = 0.009]. There was a significant interaction of Congruence × Stimulation [*F*(1,55) = 5.321, *p* = 0.025, η_p_^2^ = 0.088] and Block × Congruence × Stimulation [*F*(1.72,110) = 3.900, *p* = 0.029, η_p_^2^ = 0.066]. Follow-up estimation plots (Figure 4A) indicated higher accuracy for congruent cards in block 2 for the active tDCS group (*d* = 0.563, 95% CI [0.170, 0.961]). At the same time, these participants responded worse for incongruent cards in this block, albeit this effect was less convincing (*d* = −0.352, 95.0% CI [−0.734, 0.020]). No transfer effects from block 2 to block 3 were observed, as on the third block, there was no Stimulation effect for both congruent (*d* = 0.113 95.0% CI [−0.254, 0.498]) and incongruent cards (*d* = −0.120, 95.0% CI [−0.481, 0.264]).

We also found a significant Congruence × Valence × Stimulation interaction [*F*(1,55) = 4.821, *p* = 0.032, η_p_^2^ = 0.081]. Estimation plots revealed higher accuracy on congruent winning cards (Go-to-Win) for the active HD-tDCS vs. the sham group (*d* = 0.421, 95% CI [0.107, 0.738]), and a tendency toward lower accuracy for incongruent winning cards (NoGo-to-Win; *d* = −0.274, 95% CI [−0.583, 0.024]; Figure 4B). The two groups’ performance were similar on Avoid cards (congruent NoGo-to-Avoid cards: (*d* = 0.167, 95% CI [−0.144, 0.476]; incongruent Go-to-Avoid cards: (*d* = 0.037, 95% CI [−0.268, 0.338]).

Other effects were not significant or were of secondary interest because they did not include the effect of Stimulation. Results of the rmANOVA for all interactions in response accuracy are shown in Supplementary Table 3 of the Supplementary material.

#### 3.1.5 EEG results

**Figure 5:**
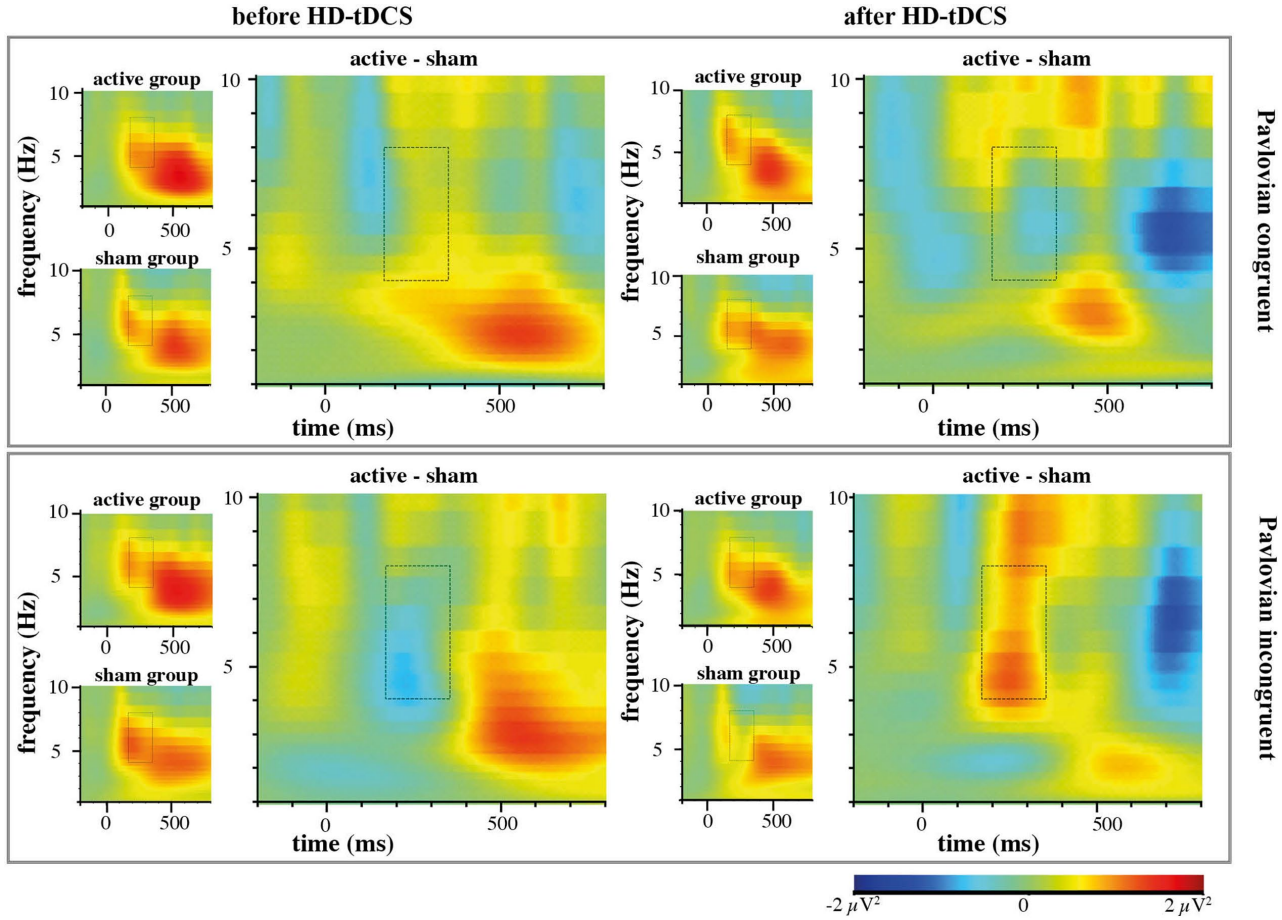
EEG time-frequency analysis of group contrasts separately for Pavlovian congruent and incongruent cards with highlighted region of interest in Theta frequency (4-8 Hz) 175–350 ms after stimulus onset.

**Figure 6:**
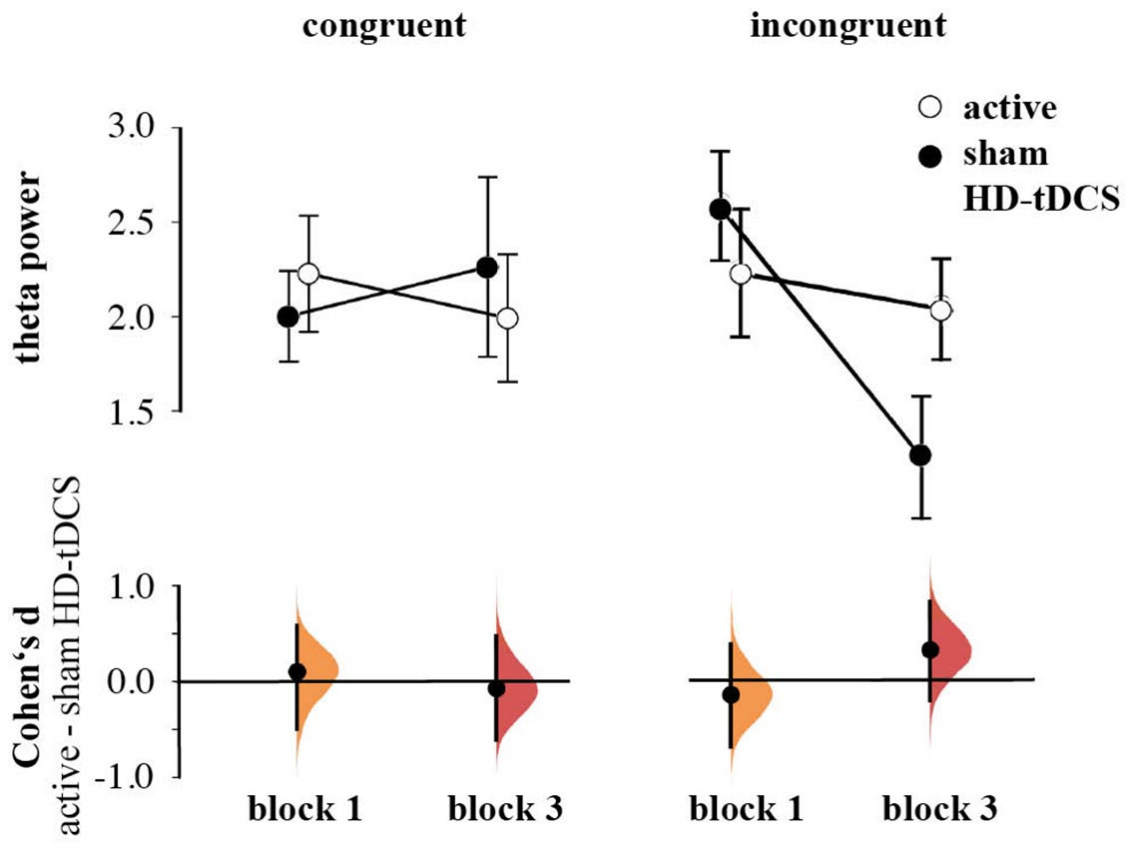
Means and SEs of the Theta power by group, congruence, and block. Cumming plots of Cohen’s d of differences between active and sham HD-tDCS groups.

In contrast to our hypothesis regarding stronger anticipated MFθ power for the active HD-tDCS group in Blocks 2 and 3, we found no significant effects of Stimulation, Block, or Congruency. Instead, there was a tendency toward the three-way interaction between Block × Congruence × Stimulation [*F*(1,53) = 3.259, *p* = 0.077, η_p_^2^ = 0.007], predominantly arising from enhanced MFθ in the active HD-tDCS group post-stimulation (Figure 5), but this effect was not convincing as the 95% CI estimate for Cohen’s *d* included zero (*d* = 0.317, 95% CI [−0.206, 0.820]; Figure 6). For further elucidation, see the time-frequency plots (Figure 5) showing the tendency toward a Stimulation effect on Pavlovian-incongruent cards.

All results of the rmANOVA for MFθ power are shown in Supplementary Table 4 of the Supplementary material.

### 3.2. Model-based analysis

**Figure 7:**
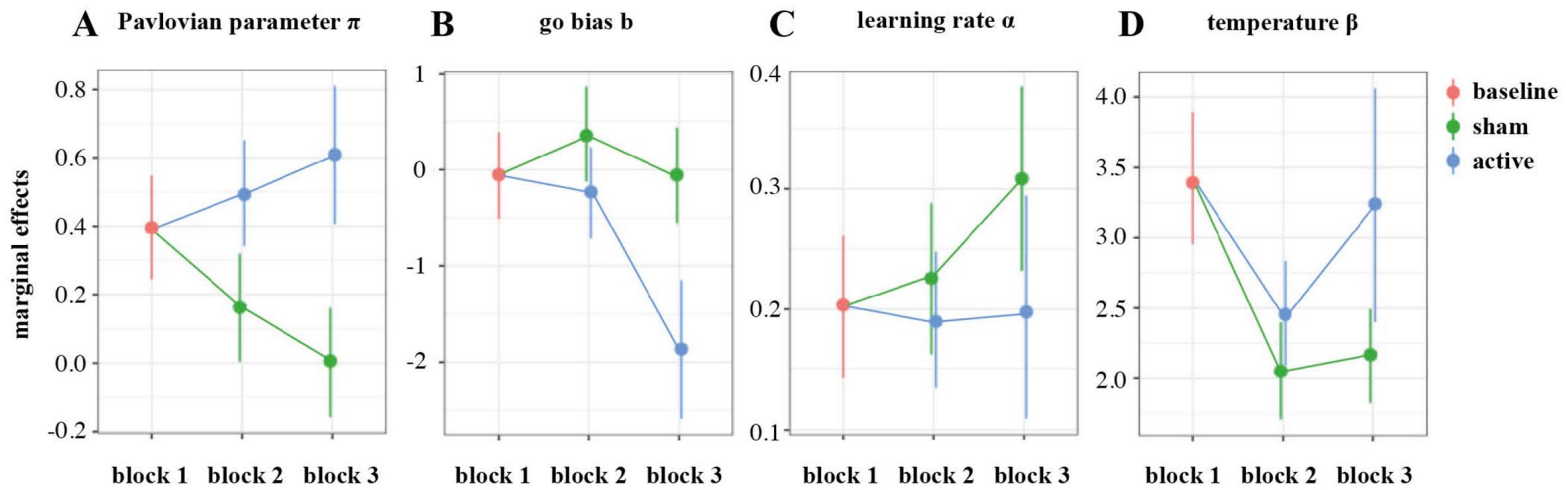
Marginal effects of group on model-based parameters during blocks 2 and 3 in comparison to the first block: Pavlovian parameter *π* **(A)**, go bias *b* **(B)**, learning rate *α* **(C)**, and temperature *β* **(D)**.

Relative to the first block, the Pavlovian parameter π decreased relevantly with loss of control in block 2 (MC = −0.233, 95% HDI [−0.302, −0.157], ER^⊖^ = ∞), and the same pattern carried over to the third block (MC = −0.389, 95% HDI [−0.465, −0.313], ER^⊖^ = ∞). This effect was counteracted by HD-tDCS, leading to an increase in π during (MC = 0.329, 95% HDI [0.233, 0.427], ER^⊕^ = ∞) and following stimulation (MC = 0.273, 95% HDI = [0.170, 0.380], ER^⊕^ = ∞; Figure 7A). Thus, in contrast to PPI, the model-based Pavlovian parameter not only increased during active HD-tDCS, but also showed a transfer effect in block 3. In order to further investigate if the change in PB from block 1 to block 3 in the active vs. sham HD-tDCS groups was associated with modulations on MFθ, we performed mixed-effects regression analysis with Stimulation, Block, MFθ and their interactions as fixed effects, participants as random effect and parameter π as an outcome variable. While this analysis confirmed the increase in PB from pre- to post-stimulation in the active HD-tDCS group only, this effect was not mediated by MFθ power (see the Supplementary Table 5 and Supplementary Figure 1 for comprehensive results).

The loss of control condition led to an increased go-bias (b) parameter (MC = 0.406, 95% HDI [0.092, 0.721], ER^⊕^ = 161). This effect did not carry over to the third block (MC = −0.00260, 95% HDI = [−0.321, 0.329], ER^⊖^ = 1.02). The active HD-tDCS group showed substantially lower go-bias during (MC = −0.585, 95% HDI [−0.993, −0.144], ER^⊖^ = 299) and after stimulation (MC = −1.23, 95% HDI [−1.65, −0.792], ER^⊖^ = ∞; Figure 7B).

The learning rate parameter α did not change under loss of control (MC = 0.0765, 95% HDI [−0.0581, 0.226], ER^⊕^ = 5.81), but increased in the third block (MC = 0.330, 95% HDI [0.181, 0.488], ER^⊕^ = ∞). There was a tendency toward a decrease under active HD-tDCS in block 2 (MC = −0.126, 95% HDI [−0.316, 0.0561], ER^⊖^ = 9.60) and a clear reduction in comparison to sham stimulation during block 3 (MC = −0.233, 95% HDI [−0.474, −0.00662], ER^⊖^ = 41.6; Figure 7C).

The temperature parameter β decreased under loss of control (MC = −0.504, 95% HDI [−0.633, −0.367], ER^⊖^ = ∞) and remained low on block 3 (MC = −0.448, 95% HDI [−0.571, −0.322], ER^⊖^ = ∞). The reduction in parameter β was less pronounced under active HD-tDCS (MC = 0.180, 95% HDI [0.00740, 0.349], ER^⊖^ = 49.4), and was increased after stimulation in block 3 (MC = 0.216, 95% HDI [0.0396, 0.389], ER^⊕^ = 145.0; Figure 7D).

## 4. Discussion

In this study, we addressed how RL performance, particularly the relative reliance on Pavlovian vs. instrumental valuation under reduced reward/loss controllability can be influenced by HD-tDCS above the mPFC/dACC. We adopted three blocks of an orthogonalized Go-NoGo task that is sensitive to parameters of RL both in the gain and loss domains and introduced a low-controllability condition during block 2 of the task. The model-agnostic Pavlovian performance index (PPI) and the model-based Pavlovian parameter π were of primary interest for the analysis.

We found that PB was reduced during the manipulation of outcome controllability in this sample. Moreover, this effect was counteracted by active HD-tDCS, manifesting in stronger reliance on Pavlovian valuation under the loss-of-control condition, with a prolonged (transfer) effect for the Pavlovian parameter π when control over feedbacks was regained. Finally, HD-tDCS did not influence MFθ power, nor did we find an association between changes in PB from pre- to post-stimulation and that of MFθ.

These results were surprising, as initially, we expected our controllability manipulation of the Go/NoGo task to induce changes in choice behavior that resemble LH. Following the argument put forward by Maier and Seligman (15), we postulated that the behavior associated with LH might be driven by automatic response tendencies, and that such bias in action selection arises from the Pavlovian valuation system (10, 14). Moreover, loss of control led to an increased reliance on the Pavlovian valuation system in previous studies (10, 14). Therefore, we expected a similar result in block 2 with sham HD-tDCS.

Within the “learned controllability” framework (15), once agents learn that they have control over environmental events, top-down mechanisms are triggered to suppress the expression of LH-like states. Conversely, the absence of control over outcomes would be associated with stronger Pavlovian bias. Since we expected that HD-tDCS above the mPFC/dACC would suppress the Pavlovian response tendency (reflected in an increased MFθ power) under Pavlovian-instrumental conflict (NoGo-to-Win and Go-to-Avoid trials), we also anticipated weaker PB both during and following our controllability manipulation in the active HD-tDCS group. These results would have provided direct evidence for the learned controllability view of LH. However, in the current study, we did not see this effect.

### 4.1. Reduced outcome controllability does not necessarily increase Pavlovian bias

Findings from the sham group are at odds with previously reported data (10, 14). Both of these studies reported stronger PB during periods of reduced outcome controllability. However, the RL task by Dorfman and Gershman (10) focused on the gain domain without any loss trials and implemented a more subtle controllability manipulation with “decoy trials.” The task structure in Csifcsák et al. (14) was identical to the one used here, but the controllability manipulation was implemented differently. The previous study created “control-yoked” pairs of participants, with the yoked group being presented with intermittent absence of outcome controllability. Notably, that protocol provided matching reward/loss frequencies between the two groups, which led to an “illusion of control” as yoked participants did not report weaker perceived controllability at the end of the task. In the current study, we manipulated both the stimulus-outcome contingency and the reward frequency by setting the contingency level to 50%. Thereby, we created a more salient manipulation in block 2, resulting in lower scores for self-reported reward/loss controllability by our participants.

Recently, it has been argued that behavior might be more sensitive to “subjective” rather than “objective” controllability. In states with overestimated perceived controllability (illusion of control), agents utilize choice strategies distinct from those with low perceived control (11). Our finding of PB steadily reducing throughout the task from block 1 to block 3 in the sham group resembles the pattern typically seen in humans when outcomes are controllable (4, 14, 24). Thus, it is possible that whether participants adopt different choice strategies, depend on whether the subjective level of control over feedbacks aligns with objective controllability. The relatively low reward and high loss rates seem to have prevented our participants from developing an illusion of control, and this might have led to the different pattern of PB change under loss of control in comparison to the previous studies.

In addition to failing to find enhanced reliance on the Pavlovian valuation system in the sham group, we also found no transfer effect. Neither the response accuracy nor the self-reported outcome controllability was compromised in block 3 compared to block 1. Finally, our controllability manipulation under sham HD-tDCS could not capture the core features of LH, which is also evident from the model-based go-bias parameters: One characteristic feature of LH would have been a reduced go-bias following loss of control, depicting behavioral passivity and reduced motivation to explore the environment. However, the go-bias parameter remained largely unchanged throughout the task under sham stimulation, indicating that the tendency for action initiation was not influenced by low outcome controllability in the second block. Hence, neither subjectively nor objectively was there a sign of a lasting transfer effect in the sense of LH.

We conclude that our behavioral protocol failed to sufficiently capture aspects of LH. Future attempts at LH induction might consider going beyond manipulating action-outcome contingency alone since this approach can be confounded by outcome predictability rather than being sensitive to controllability *per se* (34–36). In addition, manipulating reward/loss frequency more intensively should be considered since this feature was found to be more potent than contingency manipulation in reducing exploratory behavior in a different LH-induction paradigm (25).

### 4.2. HD-tDCS above the mPFC/dACC induces LH-like behavior during and following low outcome controllability

Our key finding is that active HD-tDCS led to a stronger Pavlovian bias during and following manipulations of outcome controllability. While we expected this effect to be in the other direction (i.e., active stimulation reduces PB), our result points to a role of mPFC/dACC in dynamic adjustments of choice strategies during RL (37). In line with this argument, mPFC/dACC and related network structures, such as the caudate nucleus and the posterior parietal cortex, have been implied in the parsing of action-outcome contingency (38).

Apart from an increase in PB during and after the loss of control, active HD-tDCS led to a reduced go-bias, which is of particular interest, since both enhanced PB and reduced go-bias have been previously linked to LH (10, 14, 15). In a previous study with a pairwise controllability manipulation schedule (yoking) but using exactly the same HD-tDCS protocol, the authors reported better overall response accuracy following HD-tDCS when control over feedbacks was regained (26). In the current study, task performance was not affected, perhaps compensated for by an increase in exploration (higher temperature). The model-based analysis further showed a stable, relatively low learning rate when control was regained. In other words, the participants showed less active behavior and were slower at incorporating new information, which is in accordance with the notion that active HD-tDCS promoted LH-like behavior.

This interpretation of results is in line with earlier work implicating subregions of the mPFC/ACC in LH (19, 39). While speculative at this point, future research is warranted on clarifying the role of this cortical area in behavior resembling LH, and in particular, to test if inhibitory stimulation of the mPFC/dACC with cathodal HD-tDCS or repetitive transcranial magnetic stimulation (rTMS) protocols such as low-frequency or continuous theta-burst stimulation can produce effects in the opposite direction.

Any attempt to interfere with the onset of LH-like behavior will have the potential to uncover interventional techniques that can also be tested in clinical populations with LH symptoms (11). The discrepancy between the current study and the study by Csifcsák and colleagues (26) can be attributed to how the loss of control was consciously perceived by study participants, as well as to how PB was modulated by HD-tDCS. The pairwise yoking protocol in the previous study was not explicitly recognized by participants, and was accompanied by somewhat weaker PB and corresponding changes in response accuracy for win (but not avoid) cards only. Our controllability manipulation was clearly recognized in block 2, and active HD-tDCS led not only to stronger PB but also to more optimal responses to congruent vs. incongruent trials in the gain domain. While the direction of the HD-tDCS effect differs in the two studies, it is noteworthy that both indicate a stronger association between mPFC/dACC activity and RL in reward-predictive trials, while responding to cards signaling potential losses was less affected, see also (40).

### 4.3. Is LH-like behavior during RL related to the suppression of Pavlovian response tendencies?

We cannot rule out the possibility that increased PB during HD-tDCS was driven by MFθ power in block 2, since we could not directly investigate this due to stimulation artifacts. However, the fact that the change in PB from block 1 to block 3 was not associated with modulations of MFθ across these blocks suggests that there was no involvement of this type of top-down control. This contradicts the learned controllability view of LH, which posits that under low outcome controllability, suboptimal coping strategies and behavioral passivity are causally linked to activity in subcortical nuclei that are not under (strong) top-down inhibition from the mPFC (15). Given our results on HD-tDCS-related increase in PB, the learned controllability view of LH could have been supported by finding weaker MFθ in block 3 following real HD-tDCS and/or a significant negative association between MFθ and the block-wise change in the model-based Pavlovian parameter.

However, two important aspects of the current pattern of results should also be highlighted that prevent us from drawing conclusions on the plausibility of the learned controllability framework. First, under sham stimulation, PB was not influenced by reduced outcome controllability, undermining the utility of the current controllability manipulation in inducing LH-like behavior (see discussion 4.1). Second, active mPFC/dACC stimulation did not influence MFθ, which is a puzzling finding given that this oscillatory response has been repeatedly linked to mPFC/dACC activity (24). The precise function of MFθ has been called into question by a recent study suggesting that MFθ reflects cue-specific valence monitoring and corresponds to evidence accumulation for an action being worth the effort (41). An alternative regulatory system has been proposed by a recent study (42), which showed catechol-o-methyltransferase inhibitor tolcapone to globally decrease Pavlovian bias, pointing to cortical dopaminergic top-down regulation. In future studies, frequency-specific methods, such as transcranial alternating current stimulation (tACS) or rTMS, might be better suited to disambiguate the precise function of MFθ in Pavlovian-instrumental arbitration.

Altogether, the assumption that behavioral signatures of LH might be linked to weak top-down control associated with changes in MFθ awaits validation from future studies. However, we have provided novel insight into the neural mechanisms of the arbitration between Pavlovian vs. instrumental valuation, notably, that PB can be enhanced via excitatory non-invasive stimulation of the mPFC/dACC, but without concomitant changes in MFθ (in contrast to previously reported findings) (14, 17, 23). Furthermore, we have found that stimulation of the mPFC/dACC can also modulate other latent parameters of RL (go-bias, learning rate, and randomness of choice) without compromising overall task performance.

### 4.4. Limitations

This study has several limitations. First, with the current design, we did not distinguish between inducing feelings of uncontrollability and feelings of failure. However, this distinction can be relevant for human psychopathology. See Pryce et al. (16) for further discussion of the clinical importance of helplessness and hopelessness. Second, we did not assess any long-term effects of HD-tDCS on PB. Given that current clinical applications of noninvasive brain stimulation methods routinely use multiple sessions and expect longer lasting effects, an assessment of long-term effects of HD-tDCS over the mPFC/dACC on basal learning mechanisms should be the subject of further studies. Third, our study focuses on operant conditioning with a Pavlovian-valuation component and therefore, the current results cannot be generalized to studies on pure classical conditioning. However, there is evidence that the mPFC/dACC might be involved in some aspects of classical conditioning/extinction paradigms in humans, both in the appetitive and aversive domains (43–45). Finally, in line with previous studies (14, 26), we focused on a narrow age-range of healthy adults from the university community. Therefore, our results do not necessarily generalize to older adults or clinical populations.

## 5. Conclusion

In this study, we found a progressive decrease of Pavlovian bias during and after the loss of control over feedback on a Go-NoGo RL task. This effect was counteracted by active HD-tDCS over the mPFC and dACC without affecting MFθ. The results were at odds with previous findings reporting LH-like behaviors under and after the loss of control. The discrepancy possibly arose due to changes in the task design. In this study, we manipulated not only the action-outcome contingency (as in previous studies) but also the reward frequency, which led to the participants being aware of the lack of control over feedback.

As slight changes in the paradigm seem to deliver conflicting results in different studies, more research into the precise mechanism of the influence of controllability manipulations on RL, as well as the role of MFθ-associated top-down inhibition in controllability attribution, is needed. Furthermore, the role of mPFC/dACC in behaviors resembling LH should be further explored using other intervention modalities. Likewise, the link between LH and weak top-down control awaits validation from future studies.

## Data availability statement

The datasets presented in this study can be found in online repositories. The names of the repository/repositories and accession number(s) can be found at: https://osf.io/73huk/.

## Ethics statement

The studies involving human participants were reviewed and approved by Ethics committee of the Department of Psychology, UiT, the Arctic University of Norway. The participants provided their written informed consent to participate in this study.

## Author contributions

TS: investigation, formal analysis, and writing. LB: investigation. EM: investigation. MM: conception, methodology, analysis, writing, funding acquisition, and supervision. GC: conception, methodology, analysis, writing, and supervision. All authors contributed to the article and approved the submitted version.

## Funding

This work was supported by the Northern Norway Regional Health Authority Grant PFP1237-15 awarded to GC and MM. TS was funded by the Erasmus+ mobility program of the European Union.

## Conflict of interest

The authors declare that the research was conducted in the absence of any commercial or financial relationships that could be construed as a potential conflict of interest.

## Publisher’s note

All claims expressed in this article are solely those of the authors and do not necessarily represent those of their affiliated organizations, or those of the publisher, the editors and the reviewers. Any product that may be evaluated in this article, or claim that may be made by its manufacturer, is not guaranteed or endorsed by the publisher.
