# Supplementary material for: Transcranial direct-current stimulation enhances Pavlovian tendencies during intermittent loss of control
Source: Front Psychiatry. 2023 May 9;14:1164208. doi: 10.3389/fpsyt.2023.1164208 (PMC10203223; doi:10.3389/fpsyt.2023.1164208)
Supplement: Supplementary file 1 [file Data_Sheet_1.docx]

Supplementary Material

# Supplementary Tables

## Supplementary Table 1: Repeated measures ANOVA of within- and between-subject effects on Pavlovian performance index (PPI) for all three blocks

| \| **Effects** \| **Sum of Squares** \| **df** \| **F** \| **p** \| **η_p_²** \| \| --- \| --- \| --- \| --- \| --- \| --- \| \| **Stimulation** \| 0.270 \| 1,55 \| 4.117 \| **0.047** \| 0.070 \| \| **Block** \| 0.303 \| 1.77,97.08 \| 4.466 \| **0.017** \| 0.075 \| \| **Block ✻ Stimulation** \| 0.258 \| 1.77,97.08 \| 3.802 \| **0.031** \| 0.065 \| \|  \|  \|  \|  \|  \|  \| \| Index^1^ \| 0.020 \| 1,55 \| 2.571 \| 0.115 \| 0.045 \| \| Index ✻ Stimulation \| 0.022 \| 1,55 \| 2.814 \| 0.099 \| 0.049 \| \|  \|  \|  \|  \|  \|  \| \| **Block ✻ Index** \| 0.019 \| 1.73,95.16 \| 4.145 \| **0.024** \| 0.070 \| \| Block ✻ Index ✻ Stimulation \| 0.005 \| 1.73,95.16 \| 0.997 \| 0.363 \| 0.018 \| |
| --- | --- | --- | --- | --- | --- | --- | --- | --- | --- | --- | --- | --- | --- | --- | --- | --- | --- | --- | --- | --- | --- | --- | --- | --- | --- | --- | --- | --- | --- | --- | --- | --- | --- | --- | --- | --- | --- | --- | --- | --- | --- | --- | --- | --- | --- | --- | --- | --- | --- | --- | --- | --- | --- | --- | --- | --- | --- | --- | --- | --- |

^1^‘Index’ refers to the separate effect of reward-based invigoration and punishment-based suppression.

## Supplementary Table 2: Repeated measures ANOVA of within- and between-subject effects on Pavlovian performance index (PPI) before and after stimulation

| \| **Effects** \| **Sum of Squares** \| **df** \| **F** \| **p** \| **η_p_²** \| \| --- \| --- \| --- \| --- \| --- \| --- \| \| Stimulation \| 0.017 \| 1,55 \| 0.534 \| 0.468 \| 0.010 \| \| **Block** \| 0.155 \| 1,55 \| 7.224 \| **0.010** \| 0.116 \| \| Block ✻ Stimulation \| 0.002 \| 1,55 \| 0.072 \| 0.789 \| 0.001 \| \|  \|  \|  \|  \|  \|  \| \| Index^1^ \| 0.001 \| 1,55 \| 0.237 \| 0.628 \| 0.004 \| \| Index ✻ Stimulation \| 0.011 \| 1,55 \| 0.011 \| 0.170 \| 0.034 \| \|  \|  \|  \|  \|  \|  \| \| Block ✻ Index \| 5.200e ^-4^ \| 1,55 \| 0.321 \| 0.573 \| 0.006 \| \| Block ✻ Index ✻ Stimulation \| 0.004 \| 1,55 \| 2.334 \| 0.132 \| 0.041 \| |
| --- | --- | --- | --- | --- | --- | --- | --- | --- | --- | --- | --- | --- | --- | --- | --- | --- | --- | --- | --- | --- | --- | --- | --- | --- | --- | --- | --- | --- | --- | --- | --- | --- | --- | --- | --- | --- | --- | --- | --- | --- | --- | --- | --- | --- | --- | --- | --- | --- | --- | --- | --- | --- | --- | --- | --- | --- | --- | --- | --- | --- |

^1^‘Index’ refers to the separate effect of reward-based invigoration and punishment-based suppression.

## Supplementary Table 3: Repeated-measures ANOVA of within- and between-subject effects on accuracy

| **Effects** | **Sum of Squares** | **df** | **F** | **p** | **η_p_²** |
| --- | --- | --- | --- | --- | --- |
| Stimulation | 0.056 | 1,55 | 0.509 | 0.478 | 0.009 |
| **Block** | 10.264 | 1.79,98.42 | 93.274 | \| **< .001** \|  \| \| --- \| --- \| | \| 0.629 \|  \| \| --- \| --- \| |
| Block ✻ Stimulation | 0.039 | 1.79,98.42 | 0.350 | 0.682 | 0.006 |
|  |  |  |  |  |  |
| **Congruence** | 2.384 | 1,55 | 22.547 | **< .001** | 0.291 |
| **Congruence ✻ Stimulation** | 0.563 | 1,55 | 5.321 | **0.025** | 0.088 |
|  |  |  |  |  |  |
| Valence | 0.002 | 1,55 | 0.079 | 0.780 | 0.001 |
| Valence ✻ Stimulation | 6.295e^-4^ | 1,55 | 0.020 | 0.887 | 3.712e^-4^ |
|  |  |  |  |  |  |
| **Block ✻ Congruence** | 0.552 | 1.72,94.47 | 4.643 | **0.016** | 0.078 |
| **Block ✻ Congruence ✻ Stimulation** | 0.463 | 1.72,94.47 | 3.900 | **0.029** | 0.066 |
|  |  |  |  |  |  |
| Block ✻ Valence | 0.020 | 1.53,84.01 | 0.292 | 0.687 | 0.005 |
| Block ✻ Valence ✻ Stimulation | 0.087 | 1.53,84.01 | 1.254 | 0.283 | 0.022 |
|  |  |  |  |  |  |
| **Congruence ✻ Valence** | 0.682 | 1,55 | 8.528 | **0.005** | 0.134 |
| **Congruence ✻ Valence ✻ Stimulation** | 0.386 | 1,55 | 4.821 | **0.032** | 0.081 |
|  |  |  |  |  |  |
| **Block ✻ Congruence ✻ Valence** | 0.280 | 1.94,106.94 | 3.899 | **0.024** | 0.066 |
| Block ✻ Congruence ✻ Valence ✻ Stimulation | 0.148 | 1.94,106.94 | 2.060 | 0.134 | 0.036 |

## Supplementary Table 4: Repeated measures ANOVA of within- and between-subject effects on Theta power

| **Effects** | | **umo f Squares** | | **df** | | **F** | **p** | | | **η²** |  |
| --- | --- | --- | --- | --- | --- | --- | --- | --- | --- | --- | --- |
| Group |  | 0.506 |  | 1,53 |  |  | 0.030 |  | 0.864 | 3.612e^-4^ |  |
|  |  |  |  |  |  |  |  |  |  |  |  |
| Block |  | 7.659 |  | 1,53 |  |  | 2.380 |  | 0.129 | 0.005 |  |
| Block ✻ Group |  | 1.419 |  | 1,53 |  |  | 0.441 |  | 0.510 | 0.001 |  |
|  |  |  |  |  |  |  |  |  |  |  |  |
| Congruence |  | 0.530 |  | 1,53 |  |  | 0.186 |  | 0.668 | 3.784e^-4^ |  |
| Congruence ✻ Group |  | 0.778 |  | 1,53 |  |  | 0.273 |  | 0.604 | 5.556e-4 |  |
|  |  |  |  |  |  |  |  |  |  |  |  |
| Block ✻ Congruence |  | 8.216 |  | 1,53 |  |  | 2.915 |  | 0.094 | 0.006 |  |
| Block ✻ Congruence ✻ Group |  | 9.187 |  | 1,53 |  |  | 3.259 |  | 0.077 | 0.007 |  |

## Supplementary Table 5: Linear mixed-effect analysis of Stimulation, Block, and midfrontal Theta power on the model-based Pavlovian parameter π

| **Fixed Effects Estimates** | | | | | | | | | | | |
| --- | --- | --- | --- | --- | --- | --- | --- | --- | --- | --- | --- |
| **Effect** | | **Estimate** | | **SE** | | **df** | | **t** | | **p** | |
| **Intercept** |  | 0.609 |  | 0.019 |  | 52.970 |  | 31.760 |  | **< .001** |  |
| **Stimulation** |  | -0.063 |  | 0.019 |  | 1, 52.97 |  | -3.295 |  | **0.002** |  |
| **Block** |  | 0.018 |  | 0.002 |  | 1,49 |  | 11.400 |  | **< .001** |  |
| Theta |  | 0.001 |  | 0.001 |  | 1,49.57 |  | 1.368 |  | 0.178 |  |
| **Stimulation ✻ Block** |  | 0.053 |  | 0.002 |  | 1,49 |  | 32.867 |  | **< .001** |  |
| Stimulation ✻ Theta |  | -7.694e-4 |  | 0.001 |  | 1, 49.57 |  | -0.751 |  | 0.456 |  |
| Block ✻ Theta |  | 0.002 |  | 0.001 |  | 1, 49.56 |  | 1.645 |  | 0.106 |  |
| Stimulation ✻ Block ✻ Theta |  | 0.002 |  | 0.001 |  | 1, 49.56 |  | 1.499 |  | 0.140 |  |

# Supplementary Figures

## Supplementary Figure 1: Block x Stimulation effect on the model-based Pavlovian weighting parameter π

**
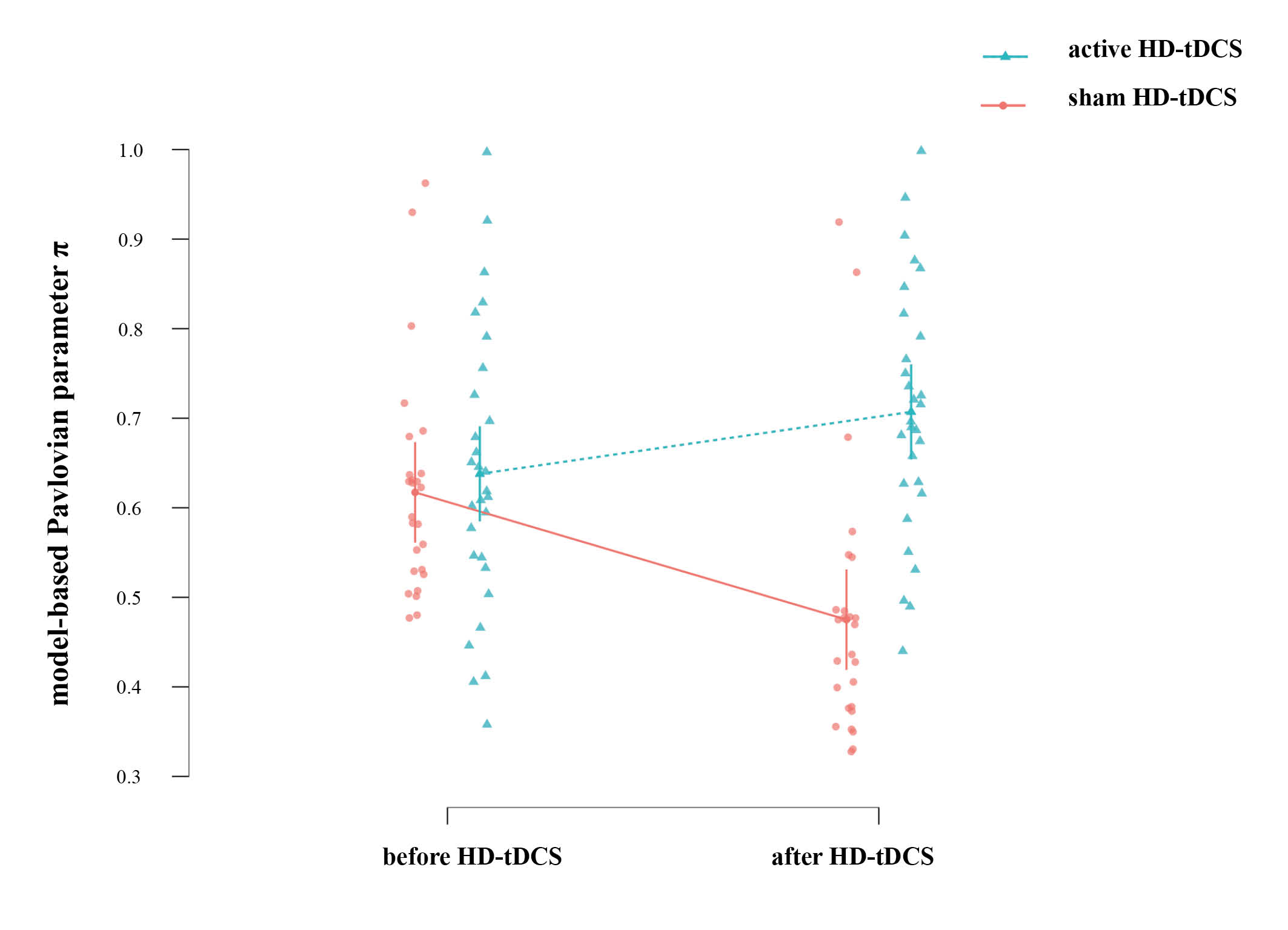
**
